# Disseminated disease caused by *Mycobacterium marseillense*: A case report and literature review

**DOI:** 10.1097/MD.0000000000035781

**Published:** 2023-10-27

**Authors:** Ji Cheng, Jun-Yan Qu, Michael R. Hamblin, Dan Hao, Xiang Wen

**Affiliations:** a Department of Dermatology, West China Hospital, Sichuan University, Chengdu, China; b Laboratory of Dermatology, Clinical Institute of Inflammation and Immunology, Frontiers Science Centre for Disease-related Molecular Network, West China Hospital, Sichuan University, Chengdu, China; c Centre of Infectious Diseases, West China Hospital, Sichuan University, Chengdu, China; d Laser Research Centre, Faculty of Health Science, University of Johannesburg, Doornfontein, South Africa.

**Keywords:** case report, clinical manifestation, disseminated disease, *Mycobacterium avium* complex, *Mycobacterium marseillense*

## Abstract

**Rationale:**

Among numerous types of nontuberculous mycobacterial infections, *Mycobacterium avium* complex is a related group of species, which can cause various diseases in humans. *Mycobacterium marseillense* is a member of the *Mycobacterium avium* complex, which accounts for only a small proportion of species, but causes rare diseases affecting the lungs, lymph nodes, skin, and tendon sheath. So far, very few cases have been reported.

**Patient concerns:**

A 76-year-old male of peculiar skin infection. Metagenomic Next Generation Sequencing and bacterial culture of skin secretions revealed *M marseillense*. To the best of our knowledge, we report the first patient diagnosed with disseminated *M marseillense* infection. Here, we identified only 8 other reports of patients with *M marseillense* infection.

**Diagnoses:**

Disseminated *M marseillense* infection.

**Interventions:**

The patient was treated with clarithromycin, rifampicin, moxifloxacin, and ethambutol.

**Outcomes:**

The skin lesions of the patient showed significant improvement, and his pruritus and limb pain were notably reduced after 7 months of follow-up.

**Lessons:**

Metagenomic Next Generation Sequencing may be a useful tool to diagnose *M marseillense* infection, but the results should be confirmed by culture and mycobacterial identification.

## 1. Introduction

Nontuberculous mycobacteria (NTM) are ubiquitous opportunistic pathogens in the environment, and are responsible for the increasing worldwide incidence of diverse diseases.^[1–4]^ Among the NTM, the microorganisms of the *Mycobacterium avium* complex (MAC) are common causes of human diseases such as pulmonary infection, lymphadenitis, cutaneous infection and disseminated infection in immunocompromised patients.^[1]^ *Mycobacterium marseillense* is a newly described member species of the MAC, but only a few case reports describe it being responsible for human infections (Table 1). We report the first case where a patient was diagnosed with disseminated *M marseillense* infection using metagenomic next generation sequencing (mNGS). At the present time, *M marseillense*-associated infections are poorly understood and the optimum management is unclear. We conducted a retrospective analysis of published studies on *M marseillense* infection since its initial discovery, to summarize the clinical manifestations, diagnostic methods, treatment, and prognosis of infections.

**Table 1:** Reports of human infection with *Mycobacterium marseillense*.

Site of Infection	Age	Sex	Country	Gene Sequencing	Antibiotic therapy	Outcome	References
Pneumonia	56	M	Korea	16S rRNA, ITS, *hsp65*	None	Unknown	^[5]^
	65	M	Italy	*rpoB*, ITS	1. RFP, INH, AMK 2. LVFX, TRD, AZM 3. EB, RFP, AZM	Cured	^[6]^
Lymphadenitis	4	F	Italy	ITS	CAM, RFP + EB	Cured	^[7]^
	4	F	Greece	*hsp65*	1. CC, CTX 2. CIP, RFP, CAM	Cured	^[8]^
Skin infection	59	F	China	16S rRNA, *hsp65, rpoB*	1. RFP, INH, EB, PZA 2. CAM, RFP, EB 3. CAM, MFLX, AMK	Cured	^[9]^
	36	M	Mexico	16S rRNA, *rpoB*	1. TMP-SMZ, CAM, MFLX 2. RFP, PZA, EB, INH 3. RFP, EB	Cured	^[10]^
Tenosynovitis	73	M	US	Not reported	AZM, EB, Rifabutin	Cured	^[11]^
	85	M	Japan	*rpoB, hsp65*	1. CAM, EB, RFP 2. CAM, EB	Died from unrelated cause	^[12]^
Disseminated disease	76	M	China	mNGS	1. CAM, RFP, MFLX, AMK 2. CAM, RFP, MFLX, EB	In follow-up	This study

AMK = amikacin, AZM = azithromycin, CAM = clarithromycin, CC = clindamycin, CIP = ciprofloxacin, CTX = cefotaxime, EB = ethambutol, F = female, INH = isoniazid, LVFX = levofloxacin, M = male, MFLX = moxifloxacin, mNGS = metagenomic next generation sequencing, PZA = pyrazinamide, RFP = rifampicin, TMP-SMZ = trimethoprim-sulfamethoxazole, TRD = terizidone.

## 2. Consent

This study was reviewed and approved by the Institutional Review Board of the West China Hospital, Sichuan University. Written informed consent was secured from the patient and the next of kin of the patient for the purpose of publication of case details and images.

## 3. Case report

In November 2022, a 76-year-old man was admitted to the West China Hospital of Sichuan University, Chengdu, China, with a 2-month history of erythematous papules and subcutaneous nodules scattered over the whole body, some of which showed rupture and exudation. He denied fever, cough, sputum, pruritus, pain or other discomfort. Bone marrow aspiration in another hospital showed there were no obvious abnormalities except a slightly increased number of eosinophils. No specific treatment was given at that time. One month before hospitalization, the skin lesions described above worsened, and he developed limb pain, severe edema of lower limbs, pruritus, fatigue and fever. Subsequently, he underwent a cervical lymph node biopsy in another hospital and histological examination showed focal suppurative inflammation with abscess, granulomatous inflammation, and 1 lymph node with reactive hyperplasia. He was in otherwise good health and had no chronic disorders or history of immunosuppressive drug use. In addition, he had no definite history of insect bites or injury.

**Figure 1.**
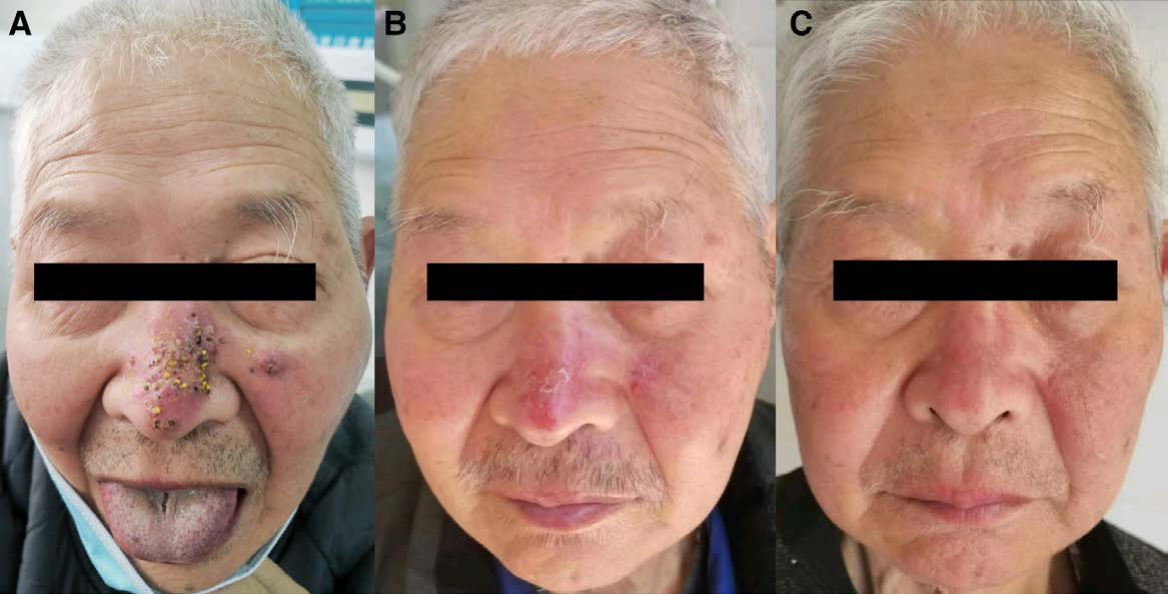
Changes in the appearance of the patient. (A) Infiltrated erythematous plaques and subcutaneous nodules with partial ulceration, exudation, and scabs on the nose. (B) The nasal skin lesions were significantly improved, with plaques flattening, and scabs shedding after four months of treatment. (C) Erythema and superficial scar on the nose after seven months of treatment.

**Figure 2.**
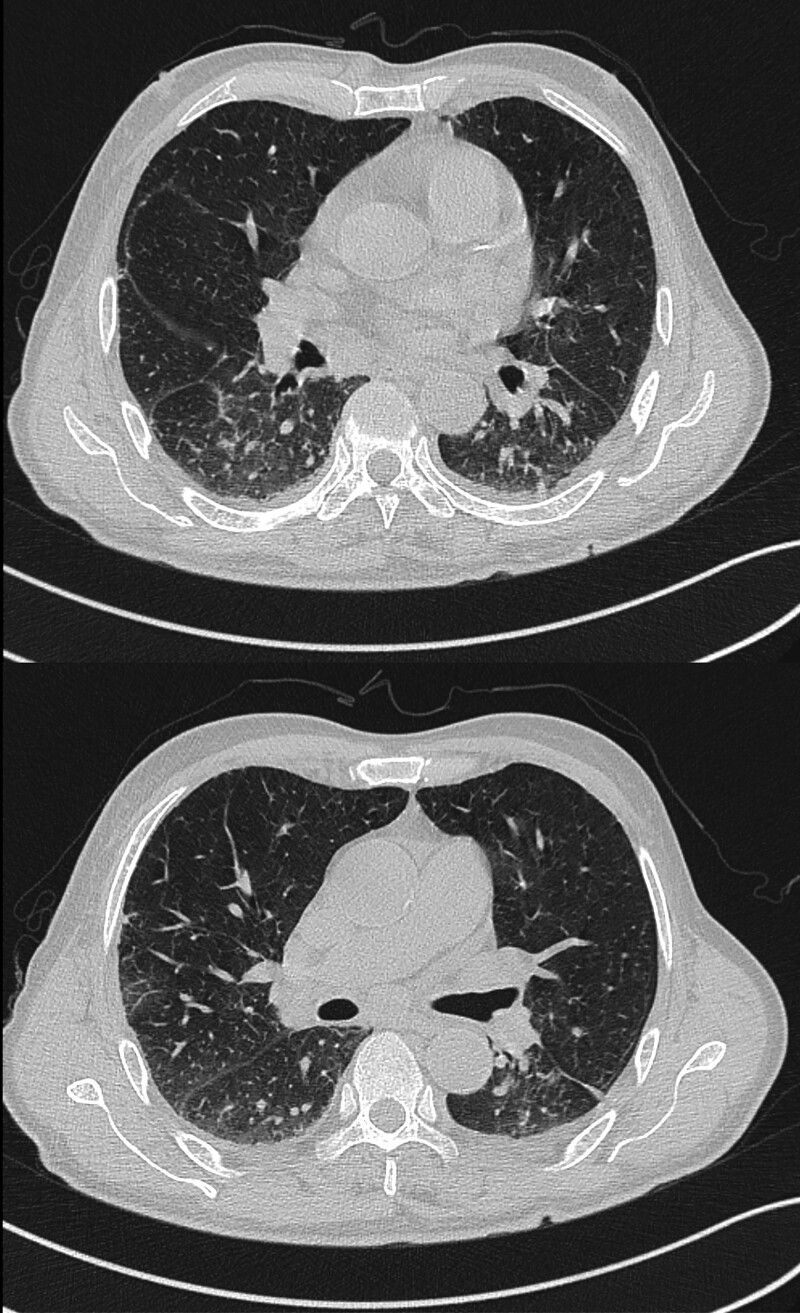
Chest computed tomographic (CT) scan. Showing multiple enlarged lymph nodes, interstitial changes with inflammation and small inflammatory nodules in both lungs.

**Figure 3.**
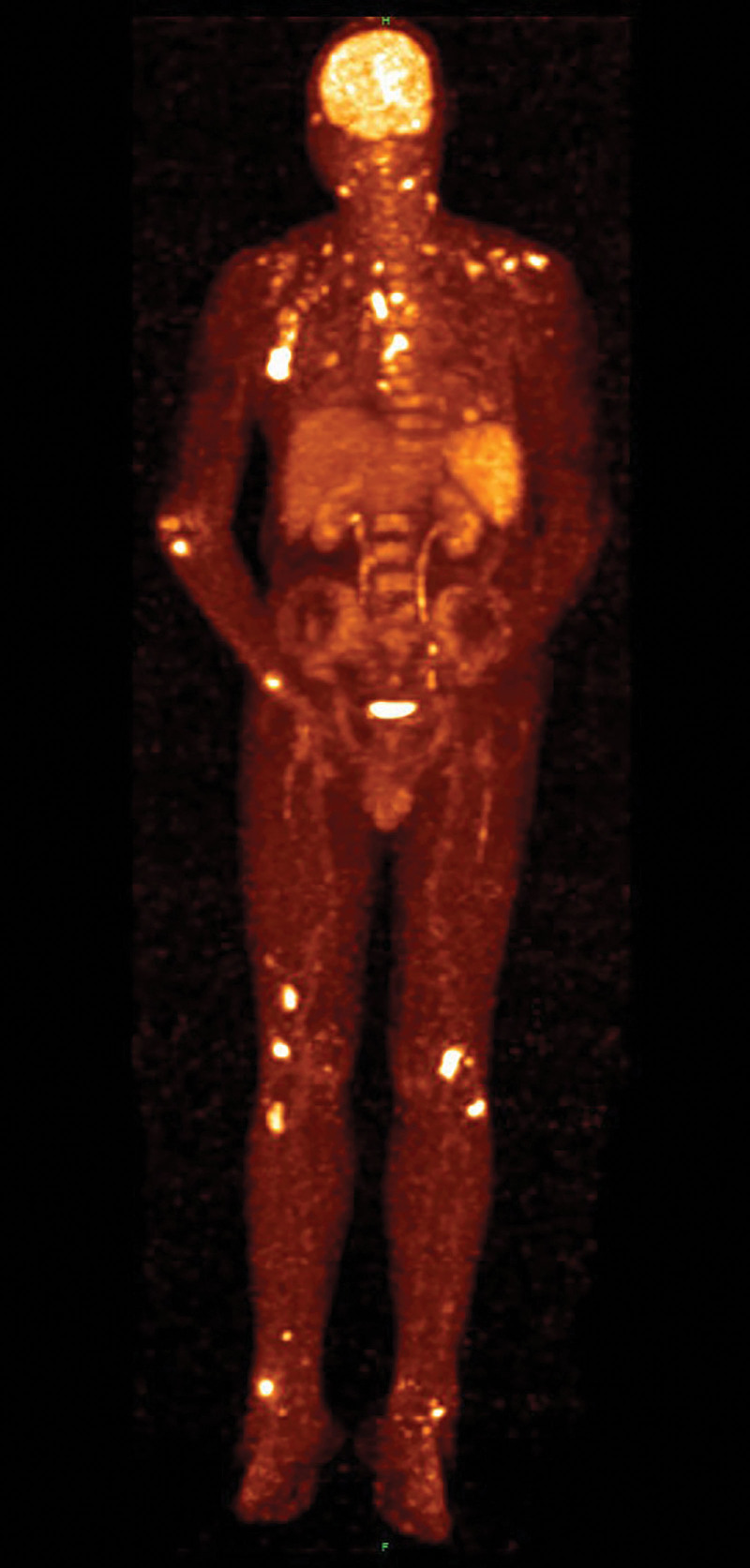
Positron emission tomography-computed tomography (PET-CT) scan. Showing an abnormal increase in glucose metabolism in cervical and thoracic lymph nodes, and in bones and soft tissues (bright spots). CT = computed tomographic.

**Figure 4.**
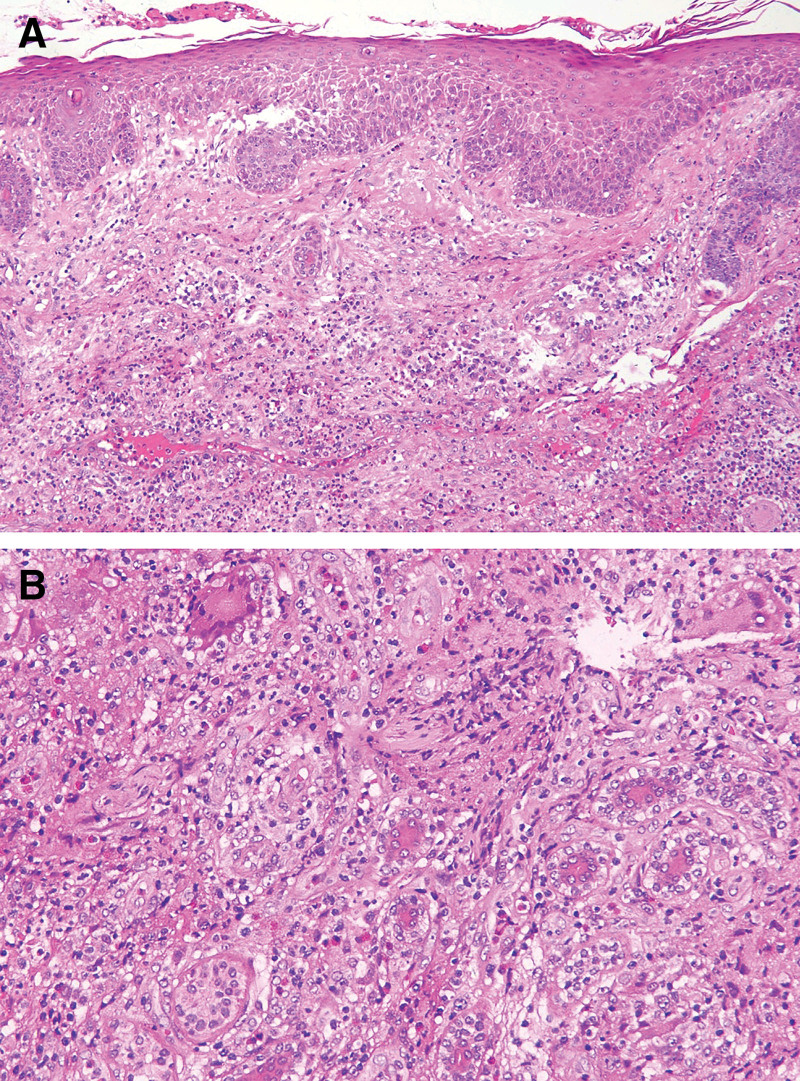
Histological examination. (A) HE × 100 showing mild hypertrophy and edema in the stratum spinosum of the epidermis. (B) HE × 200 showing epithelial granuloma with infiltration by a large number of lymphocytes, neutrophils, and a small number of eosinophils in the dermis.

On physical examination, the patient was found to have coin-sized infiltrated erythematous plaques and subcutaneous nodules with partial ulceration, exudation, and oozing of blood scattered over the whole body, especially on the nose (Fig. 1A). On medical examination, he was found to have normal respiratory sounds and enlarged lymph nodes in the axilla and groin. Laboratory testing revealed negative results for human immunodeficiency virus antibody test, aspergillus galactomannan antigen test (GM test), fungal (13)-b-D glucan test (G test), as well as negative bacterial and fungal culture of skin secretions. He had markedly elevated values of C-reactive protein, interleukin-6, procalcitonin, the corresponding results were 104.00 mg/L, 103.00 pg/mL, 0.27 ng/mL. What was more, counts of neutrophils, lymphocytes and eosinophils were entirely abnormally elevated. Epstein Barr Virus DNA (1.03 × 10^2^ copies/mL) and human cytomegalovirus DNA (<50 copies/mL) were positive. The B-lymphocyte count was moderately decreased. However, the CD4 + T-lymphocyte count remained within normal limits while the CD8 + T-lymphocyte count was slightly increased. The result of interferon gamma release assay from tuberculosis-infected T cells (TB-IGRA) was indeterminate. A chest computed tomographic (CT) scan showed multiple enlarged lymph nodes, interstitial changes with inflammation and scattered small inflammatory nodules in both lungs (Fig. 2). Positron emission tomography-computed tomographic showed an abnormal increase in glucose metabolism in multiple lymph nodes, bones and soft tissues (Fig. 3), which could suggest either a hematologic neoplasm or an infectious disease. A biopsy of facial skin tissue revealed that there was mild hypertrophy and edema in the stratum spinosum of the epidermis. There was an epithelial granuloma with infiltration of a large number of lymphocytes, neutrophils and a small number of eosinophils in the whole dermis (Fig. 4). Grocott methenamine silver, periodic acid-Schiff and acid-fast bacilli stains of the skin biopsy were all negative.

Next, nasal skin tissue biopsies were subjected to mNGS to characterize the pathogen. *M marseillense* was identified by comparison with Microbial Genome Databases. Empiric treatment was initiated using the antimicrobial drugs, clarithromycin, rifampicin, moxifloxacin and amikacin. After discharge, the amikacin was replaced with ethambutol. Two months later, the laboratory culture and identification of mycobacteria in nasal skin secretions confirmed the presence of *M marseillense*. Drug susceptibility testing revealed that the isolate was susceptible to clarithromycin, rifampicin, moxifloxacin, amikacin, imipenem, tobramycin, rifabutin and cefoxitin, but was resistant to linezolid, doxycycline, and minocycline. Based on the above clinical manifestations and laboratory testing, the patient was diagnosed with disseminated disease caused by *M marseillense*. After 7 months of follow-up, the nasal and systemic skin lesions of the patient showed significant improvement, and his pruritus and limb pain were notably reduced (Fig. 1B and C). Compared with the values at admission, C-reactive protein, interleukin-6 and procalcitonin had decreased noticeably, respectively results were 22.4 mg/L, 18.2 pg/mL, 0.14 ng/mL. Most of the abnormal indicators had returned to normal, including the counts of white blood cells, neutrophils and lymphocytes. At the present time, he is still returning for clinical follow-up examinations.

## 4. Discussion

In 2007, a statement by American Thoracic Society and the Infectious Diseases Society of America laid out the diagnostic criteria, as well as recommendations for treatment and prevention for patients suspected of having NTM disease.^[1]^ This was a step forward to recognize the importance of NTM, and has enabled clinicians to develop appropriate treatment options. Although MAC organisms had been widely isolated from the inanimate environment,^[13,14]^ our understanding of MAC, including its clear definition, was limited at that time. Initially, MAC was considered to comprise only 2 species, *M. avium* and *M. intracellulare*.^[15]^ Since then, an increasing number of studies have been conducted to identify novel species of MAC. A recent review proposed a definition for MAC, which included twelve validated published species. MAC is considered to be a group of slow-growing mycobacteria that share certain characteristics in common based on phylogenetic analysis.^[16]^ By analyzing *rpoB* and other genes, Ben Salah et al^[17]^ first isolated and identified *M marseillense* as a member of MAC.

Since the first case of lung disease caused by *M marseillense* was reported in the literature in 2014, several additional cases have gradually emerged (Table 1). Because *M marseillense* is an exceedingly infrequent and newly described mycobacterial species, only 8 cases of *M marseillense* infection have been published so far. One study reported that *M marseillense* could be isolated from patients with cystic fibrosis,^[18]^ suggesting that the incidence of *M marseillense* infection may have been underestimated, especially in cystic fibrosis patients. In this review, we analyze the published literature concerning the clinical characteristics, diagnostic methods, treatment options, and outcomes of *M marseillense* infection.

In the past, it was believed that immunocompromised patients were more susceptible to infection with MAC, especially AIDS or organ transplant patients.^[1]^ However, in the 8 reported cases of *M marseillense* infection, only 2 of them were immunocompromised, 1 with systemic lupus erythematosus and the other with renal transplantation. The remainder had no apparent immunosuppression. In our case, the patient developed disseminated *M marseillense* infection without being immunocompromised. These findings suggest that *M marseillense* infect healthy individuals, even causing disseminated infection. Except for a 37-year-old man who developed cutaneous infection by *M marseillense* after acupuncture,^[10]^ the remaining patients had no clear trigger for infection. Therefore, the route for infection and the transmission mode of *M marseillense* remain unclear. In terms of the demographic location of these 9 cases, 4 were reported in Asia, 3 in Europe, and 2 in North America. No cases have been reported in other world regions so far. It is not clear whether this is due to the geographical distribution of *M marseillense* or the limited availability of testing in other regions. The age range of infected individuals was wide, ranging from 4 to 85 years old, suggesting that the infected population was diverse. Different age groups appeared to have different clinical features. Two girls and 1 middle-aged woman were infected, while the remaining 6 patients were middle-aged or elderly men. However, this does not suggest any male to female infection ratio due to the very small sample size. The duration of the disease course varied from 2 weeks to 4 years, with most cases being relatively chronic in nature.

Currently, *M marseillense* has been associated with various clinical presentations, including lung disease,^[5,6]^ lymphadenitis,^[7,8]^ skin infections,^[9,10]^ tenosynovitis^[11,12]^ and even disseminated infection. Pulmonary disease caused by *M marseillense* typically presents with cough, occasional sputum, fever, or hemoptysis. Diffuse rales may also be detected upon auscultation, and a CT scan could reveal dense consolidation of the pulmonary lobes, diffuse nodular opacity and bronchial thickening. However, these symptoms are not specific to *M marseillense* infection, and may be present in other lung diseases. Lymphadenitis is usually characterized by a swollen mass with central fluctuation, accompanied by fever and pain in some cases. Ultrasound imaging typically shows enlarged lymph nodes with uneven echogenicity, hypoechogenic areas, and a subverted structure. The clinical manifestations of skin infection are variable and include erythematous plaques, nodules, abscesses, ulceration, fistulas, yellow scales, and crusts. Histologic examination usually reveals an inflammatory infiltrate consisting of lymphocytes, histiocytes, plasma cells, neutrophils, and some mastocytes within the dermis. Patients with tenosynovitis usually complain of an enlarged soft mass on the dorsal wrist, with or without pain. Magnetic resonance imaging typically reveals a large number of rice bodies and distension of the tendon sheath. Histological examination of the specimen often shows granulomatous inflammation, synovial hyperplasia and focal necrosis. In our case of disseminated disease caused by *M marseillense*, the patient presented with cutaneous symptoms including erythematous plaques, subcutaneous nodules and ulcers, as well as limb pain, and painless lymphadenopathy. Additionally, a color ultrasound revealed tenosynovitis in the flexor tendon of the patient’s right index finger. Based on the results of the lymph node biopsy, it is reasonable to conclude that *M marseillense* had infected his skin, tendon sheath, bone, and lymph nodes. To our knowledge, this is the first report of bone symptoms in patients with *M marseillense* infection and the first time that lymphadenitis was reported in adults.

*M marseillense* is an extremely rare and slow-growing mycobacterial species that is difficult-to identify, making the diagnosis of infections challenging. Grottola et al^[6]^ reported 1 patient who had not received a conclusive diagnosis until 6 years after the onset of symptoms. In 2009, Ben Salah et al^[17]^ proposed 3 novel MAC species including *M marseillense* by nucleic acid sequencing of the 16S rRNA gene, 16S to 23S rRNA gene internal transcribed spacer (ITS-1), as well as *rpoB*, and *hsp65* sequences. In previous patients, some mycobacterial colonies were isolated on *Lowenstein-Jensen* selective culture medium. Nevertheless, for definitive identification, they had to determine 1 or more of sequences mentioned above. In our case, we employed mNGS of skin tissue and obtained a positive result (sequence: 17,533, identification confidence: 99%) as soon as the next day after admission. To the best of our knowledge, this is the first report of the successful identification of *M marseillense* using mNGS, and underlines the potential of this method for difficult-to-detect pathogens. mNGS (also known as unbiased or clinical metagenomics) is a method in which all the nucleic acids in a sample are sequenced in parallel.^[19]^ This method allows for broadly-based pathogen detection, regardless of the type of microbe, and has even been used to discover novel organisms.^[20]^ The metagenomics sequencing of cerebrospinal fluid samples in the clinical microbiology laboratory at the University of California, San Francisco had an overall accuracy of 90% with a clinical sensitivity and specificity of 73% and 99%, respectively.^[21]^ The average success rates for pathogen detection using mNGS in clinical specimens vary between different patient populations and institutions, but are generally within a range of 50% to 70%.^[22]^ Several previous reports^[23–25]^ have shown that mNGS can detect rare pathogens that have been missed by conventional testing, making it a promising universal method for infectious disease diagnosis.

Previous drug sensitivity results have shown that *M marseillense* is generally sensitive to clarithromycin and amikacin, with a proportion of isolates also being sensitive to linezolid, moxifloxacin, or azithromycin. These results were in agreement with 2 recent in vitro antimicrobial efficacy studies, except the in vitro studies showed that *M marseillense* was sensitive to rifabutin.^[26,27]^ In 2 patients with *M marseillense* lung infections, 1 patient had antibiotic therapy suspended for other reasons, and the other patient gradually improved after 6 months of treatment with ethambutol, rifampicin, and azithromycin. Two girls diagnosed with *M marseillense* lymphadenitis were treated with antibiotics including clarithromycin, but they did not respond well, so the infected lymph nodes were removed by surgery, and then they recovered soon after. The female patient with cutaneous infection was treated with clarithromycin, rifampicin, ethambutol for 3 months, followed by clarithromycin, moxifloxacin, and amikacin for 2 months. The male patient was treated with rifampicin, pyrazinamide, ethambutol, and isoniazid for 2 months, followed by rifampicin and isoniazid for 4 months. Eventually, both of the patients were cured. A 73-year-old man with tenosynovitis was cured by taking azithromycin, rifabutin, and ethambutol for 6 months. In our case, the patient was treated with clarithromycin, rifampicin, moxifloxacin and amikacin for 1 month, and then clarithromycin, rifampicin, moxifloxacin and ethambutol for 6 months until now. Although the patient’s clinical manifestation and laboratory tests have greatly improved, it would be better if the patient could undergo a repeat Positron emission tomography-computed tomographic or chest CT for before and after comparisons. It is regrettable that this patient was not willing to undergo examinations for personal reasons. With the exception of 2 patients who discontinued treatment for other reasons and the patients diagnosed with lymphadenitis who underwent surgery, the rest of the patients recovered with long-term regular antibiotics. The combination of 2 to 4 antibiotics can achieve good efficacy, and the drugs can be adjusted according to drug sensitivity testing and treatment responses. Another key point is the adherence to medication, as treatment courses for NTM infections are often lengthy. There are a number of new drugs that may be approved in the coming years and we believe that the treatment of MAC infections will make significant progress.

## 5. Conclusion

*M marseillense* is a newly identified member of MAC, which has recently been shown to cause lung disease, lymphadenitis, skin infection, tenosynovitis, or disseminated infection. Based on previous studies and our present case, it is clear that *M marseillense* can infect multiple organ systems of the body, and its clinical manifestations are nonspecific. If conventional antibiotics are ineffective, NTM should be considered. Gene sequencing, including mNGS, may play a crucial role in the diagnosis of *M marseillense* infection and is being increasingly utilized in pathogen detection. Early diagnosis is crucial for effective treatment. Based on past treatment experience, clarithromycin is recommended. Although the treatment course is lengthy, the prognosis is optimistic. *M marseillense* infections are still rare but noteworthy. We believe that more case reports and clinical studies in the future will have a significant impact on the diagnosis and treatment of these infections.

## Author contributions

**Conceptualization:** Jun-Yan Qu.

**Formal analysis:** Dan Hao.

**Resources:** Michael R Hamblin.

**Writing – original draft:** Ji Cheng.

**Writing – review & editing:** Ji Cheng, Xiang Wen.
